# Supplementary material for: Characterization of the genomic landscape and actionable mutations in Chinese breast cancers by clinical sequencing
Source: Nat Commun. 2020 Nov 10;11:5679. doi: 10.1038/s41467-020-19342-3 (PMC7656255; doi:10.1038/s41467-020-19342-3)
Supplement: Supplementary file 1 — Supplementary Information [file 41467_2020_19342_MOESM1_ESM.docx]

**Supplementary Information**

**Characterization of the Genomic Landscape and Actionable Mutations in Chinese Breast Cancers by Clinical Sequencing**

**Supplementary Figure 1**


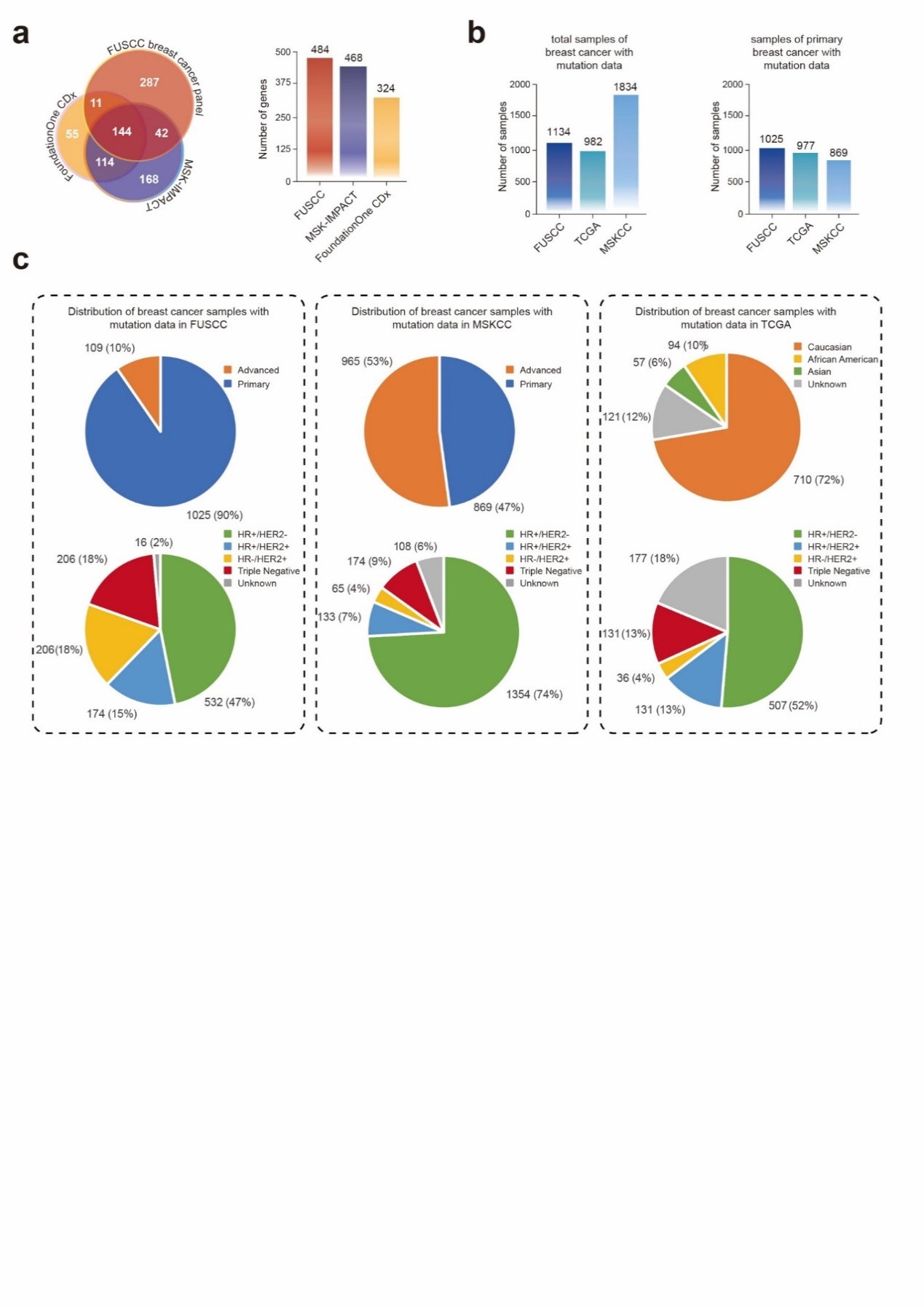


**Supplementary Fig.1丨Comparison of the sequencing panel and sample distribution with foreign open-access data.**

**a.** Venn diagrams displaying the gene panel overlap among the FUSCC, MSK-IMPACT and FoundationOne CDx breast cancer panels. The bar chart illustrates the total number of genes in the FUSCC, MSK-IMPACT and FoundationOne CDx breast cancer panels.

**b.** Total number of sequenced breast cancer samples (left) and primary breast cancer samples (right) in the FUSCC, TCGA and MSKCC datasets.

**c.** Distribution of breast cancer samples in the FUSCC (left), MSKCC (middle) and TCGA (right) datasets. The samples in the FUSCC and MSKCC datasets were grouped based on the tumor stage and molecular subtype. The samples in the FUSCC and MSKCC datasets were grouped by ethnicity and the molecular subtype.

**Supplementary Figure 2**


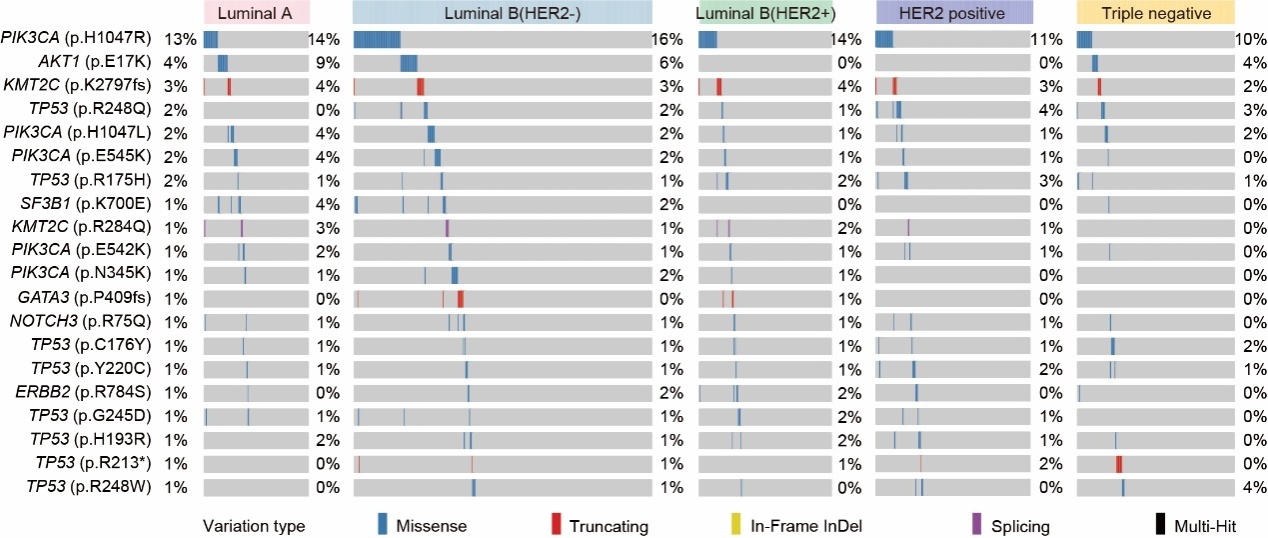


**Supplementary Fig.2丨Landscape of mutation spots ordered by the molecular subtype and mutation profile and annotated by the variation type and mutation frequency.**

**Supplementary Figure 3**


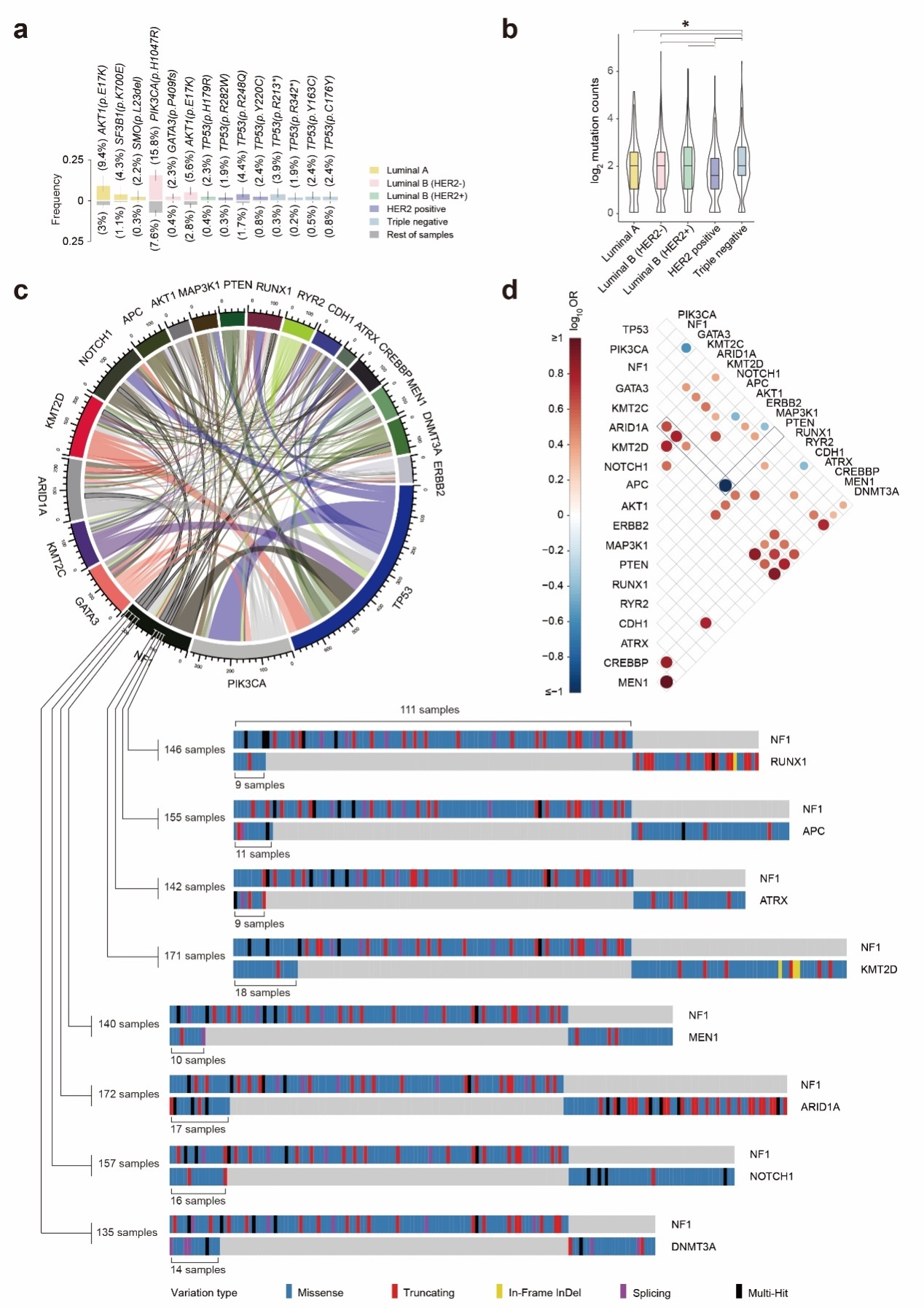


**Supplementary Fig.3丨Genomic characteristics of prospectively sequenced Chinese breast cancer.**

1. Significant enrichment of mutations spots in different subtypes of breast cancer.
2. Comparison of tumor mutation counts among different subtypes of breast cancer. Y axis: log2 (ratio of nonsynonymous mutation counts); X axis: subtype of breast cancer. *p* value is determined by Wilcoxon signed-rank test, and the asterisks indicates *p* < 0.05. The violin is bounded by the first and third quartile with a horizontal line at the median and whiskers extend to 1.5 times interquartile range (IQR).
3. Circus plot displaying the co-occurrent patterns of recurrently mutated genes in our cohort. The line thickness corresponds to the number of mutations in two co-occurrent genes. The significant co-occurrent patterns of *NF1* mutations are illustrated below.
4. Significant mutual exclusivity (blue) and co-occurrence (red) of gene mutations in our cohort. Spectrum bar: log10 (OR); the color intensity represents the scale of the value.

Source data for b are provided as a source data file.

**Supplementary Figure 4
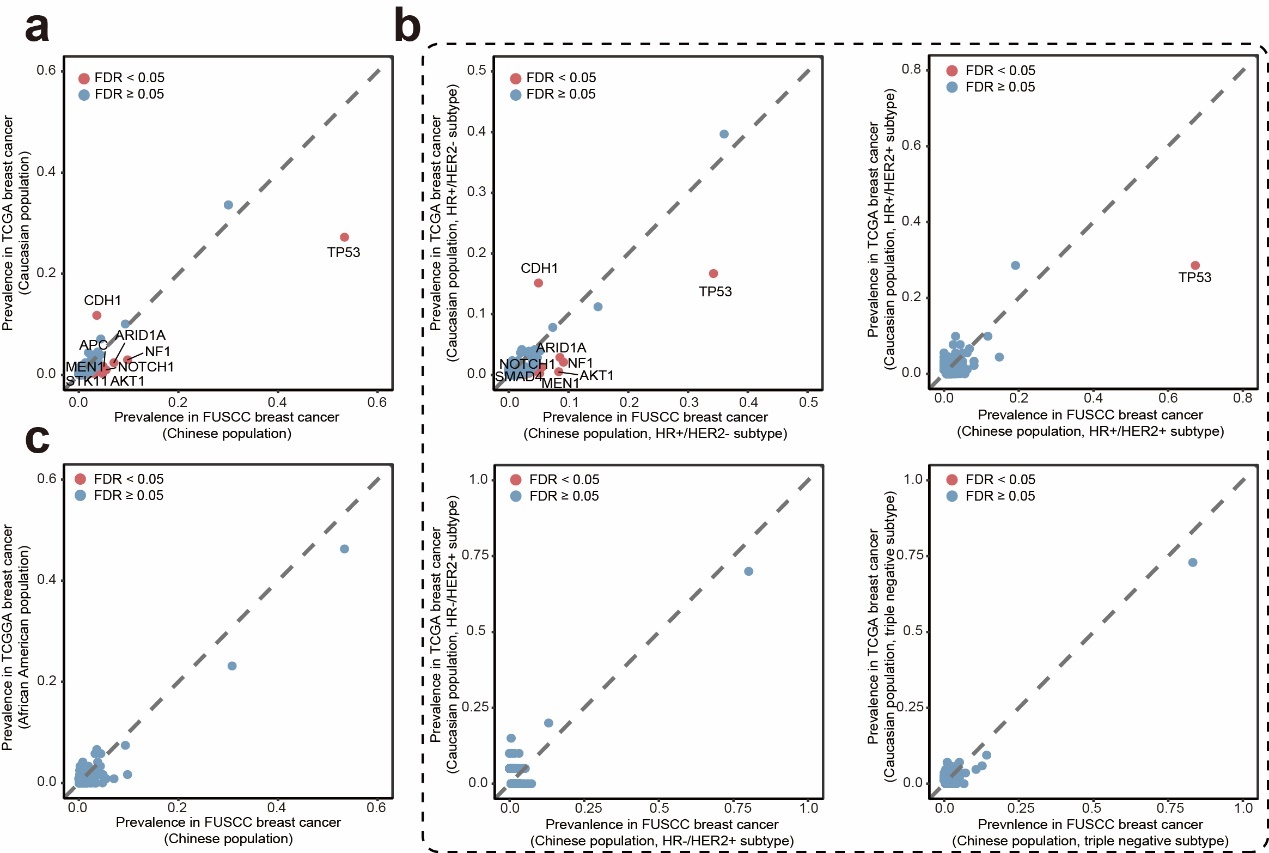
**

**Supplementary Fig.4丨Population-specific genomic mutations in Chinese breast cancer compared with TCGA data.**

1. Scatter plots of the prevalences of mutated genes in breast cancer samples from the FUSCC (x-axis) and Caucasian samples in the TCGA dataset (y-axis).
2. Scatter plots of the prevalences of mutated genes in breast cancer samples of the HR+/HER2- (top, left), HR+/HER2+ (top, right), HR-/HER2+ (bottom, left) and triple-negative (bottom, right) subtypes from the FUSCC (x-axis) and Caucasian samples in the TCGA dataset (y-axis).
3. Scatter plots of the prevalences of mutated genes in breast cancer samples from the FUSCC (x-axis) and African American samples in the TCGA dataset (y-axis).

**Supplementary Figure 5**


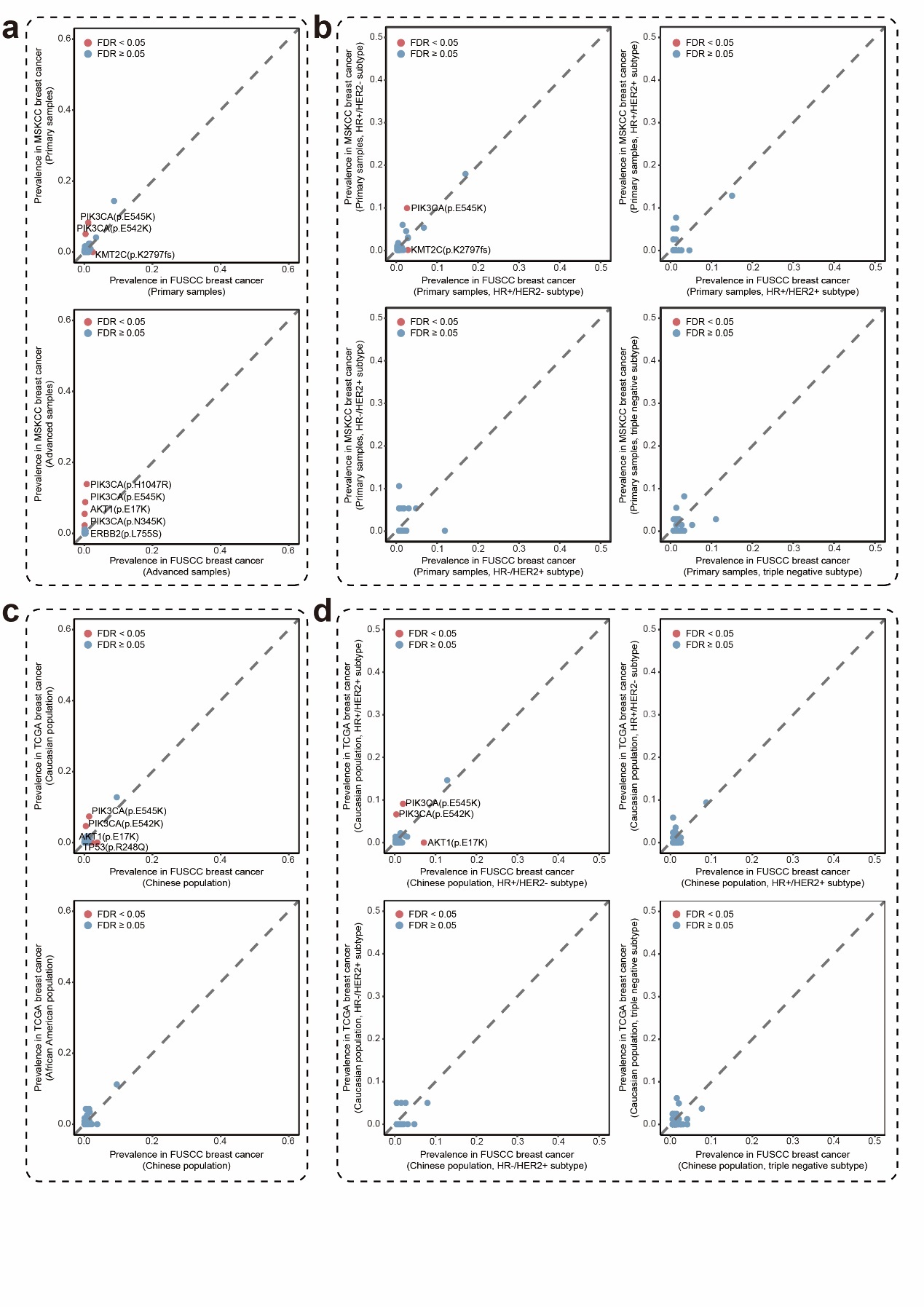


**Supplementary Fig.5丨 Population-specific mutation spots in Chinese breast cancer compared with the MSKCC and TCGA datasets.**

1. Scatter plots of the prevalence of mutation spots in primary (top) and advanced (bottom) breast cancer samples from FUSCC (x-axis) and the MSKCC dataset (y-axis).
2. Scatter plots of the prevalence of mutation spots in primary breast cancer samples of the HR+/HER2- (top, left), HR+/HER2+ (top, right), HR-/HER2+ (bottom, left) and triple-negative (bottom, right) subtypes from the FUSCC (x-axis) and MSKCC dataset (y-axis).
3. Scatter plots of the prevalence of mutated genes in breast cancer samples from the FUSCC (x-axis) and TCGA dataset (Caucasian population, top; African American population, bottom) (y-axis).
4. Scatter plots of the prevalence of mutated genes in breast cancer samples of the HR+/HER2- (top, left), HR+/HER2+ (top, right), HR-/HER2+ (bottom, left) and triple-negative (bottom, right) subtypes from the FUSCC (x-axis) and Caucasian samples in the TCGA dataset (y-axis).

**Supplementary Figure 6**
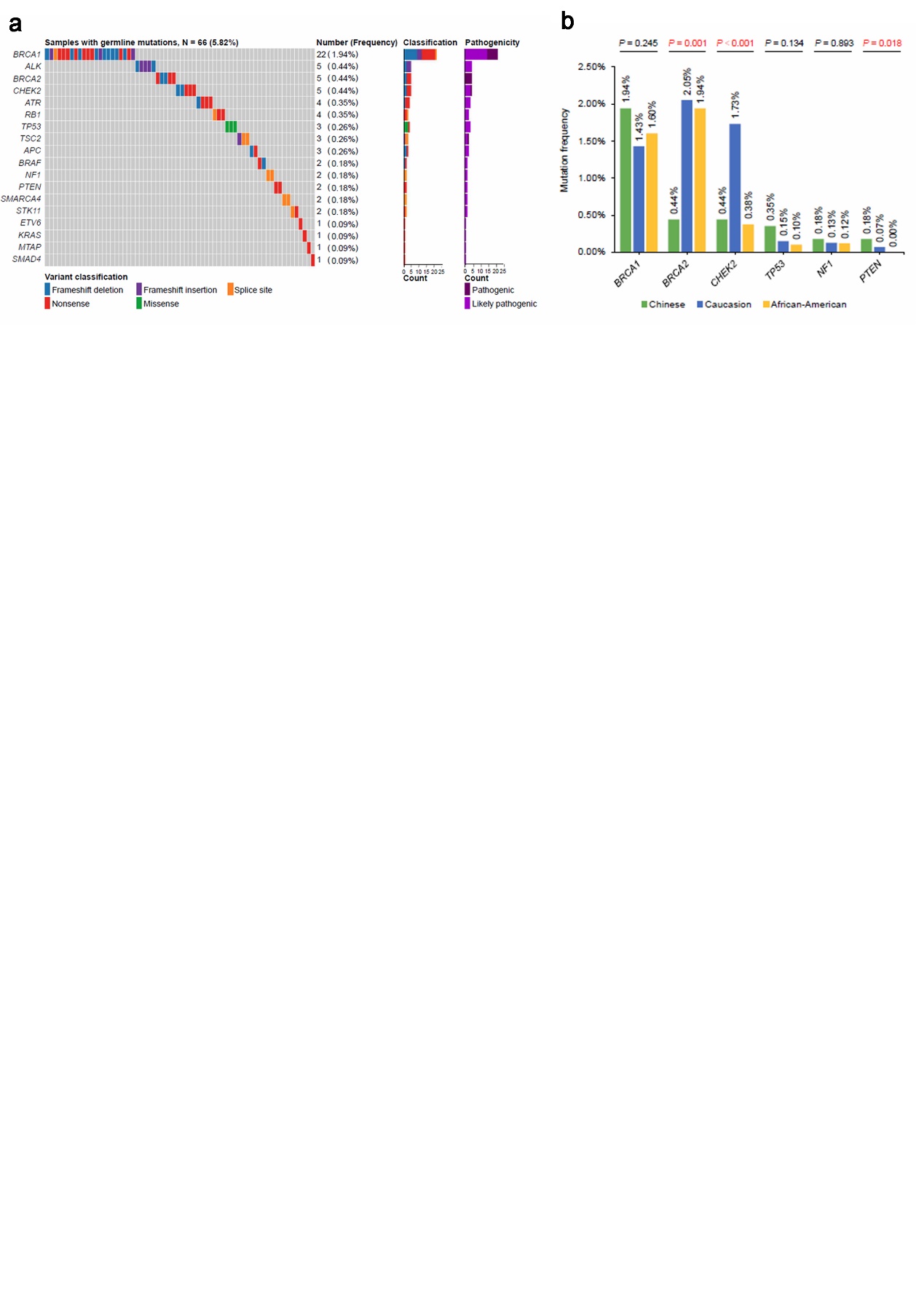
**Supplementary Fig. 6** **| Germline mutations in the FUSCC-BC cohort and comparison with Caucasian and African-American breast cancers. a.** The spectrum of germline mutations of breast cancers in our cohort. **b.** The comparison of 6 frequently mutated genes with Caucasian and African-American breast cancers (Chi-square test, two-sided).

**Supplementary Figure 7**

**
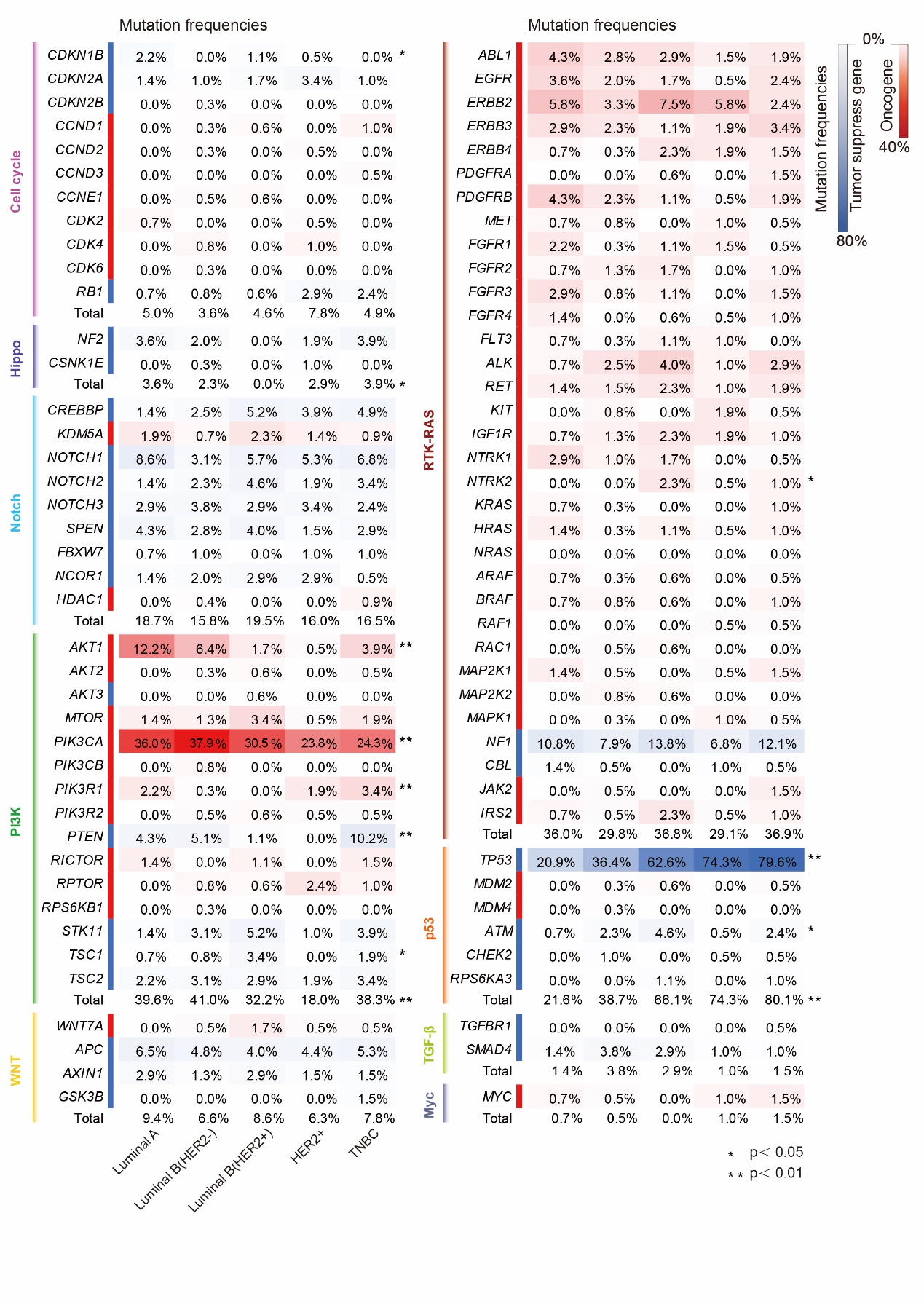
 Supplementary Fig. 7丨Heatmaps of somatic mutations in nine oncogenic signaling pathways in Chinese breast cancer.**

The mutation frequencies obtained in each gene in each oncogenic signaling pathway are displayed in the diagram. The sum of the individual gene mutation frequencies might be greater than the total of each molecular subtype within the individual pathway because some tumor samples might have multiple mutations. Red: oncogenes; blue: tumor-suppressor genes; the color intensity shows the frequency of occurrence. The last row of each pathway indicates the overall mutation frequency in the pathway in each breast cancer subtype. The asterisks indicate a statistically significant association with the subtype (Chi-square test, two-sided). Source data are provided as a source data file.

**Supplementary Figure 8**
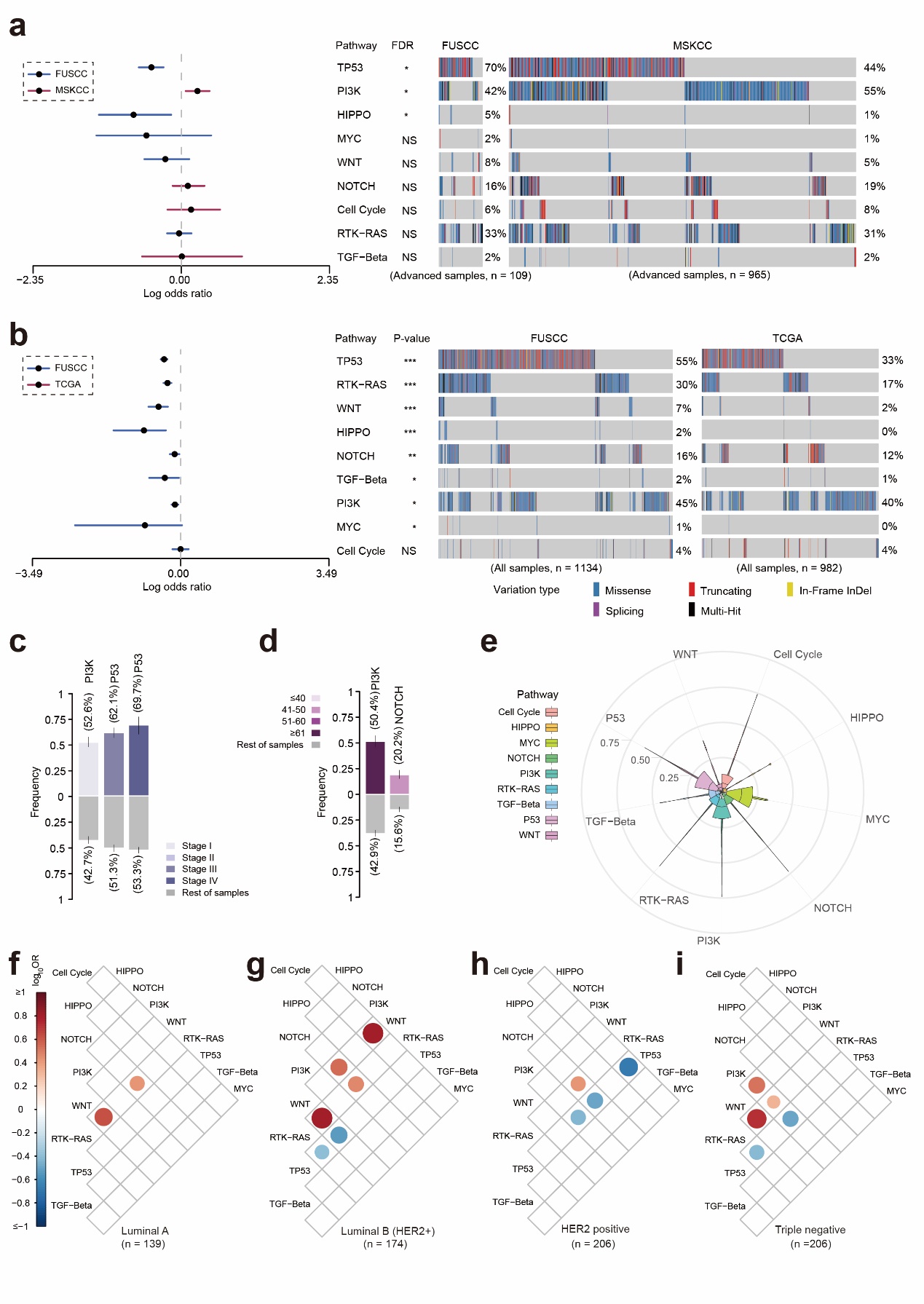


**Supplementary Fig. 8丨Characteristics of mutations in oncogenic signaling pathways in prospectively sequenced Chinese breast cancer.**

**a.** Comparison of mutations in oncogenic signaling pathways in advanced samples between our cohort and the MSKCC dataset. A total of 109 advanced breast cancer samples from FUSCC are compared with 965 advanced breast cancer samples from MSKCC by different mutation status in oncogenic signaling pathways using Fisher’s exact test, adjusted by false discovery rate (FDR). The asterisks indicate FDR < 0.05. The middle circular spot corresponds to a value for odds ratio and the lines represent 95% confidence intervals. A red or blue horizontal line represents the significant or non-significant result of the comparison of mutation frequencies between our cohort and the MSKCC’s cohort in each signaling pathway, respectively. The red line indicates a higher mutation frequency in the corresponding pathway favors in the MSKCC’s cohort, while the blue line indicates a higher mutation frequency in the corresponding pathway favors in our cohort.

**b.** Comparison of mutations in oncogenic signaling pathways between our cohort and the TCGA dataset. A total of 1134 breast cancer samples from FUSCC are compared with 982 breast cancer samples from TCGA by different mutation status in oncogenic signaling pathways using Fisher’s exact test, adjusted by false discovery rate (FDR). The asterisks indicate FDR < 0.05. the middle circular spot corresponds to a value for odds ratio and the lines represent 95% confidence intervals. A red or blue horizontal line represents the significant or non-significant result of the comparison of mutation frequencies between our cohort and the TCGA’s cohort in each signaling pathway, respectively. The red line indicates a higher mutation frequency in the corresponding pathway favors in the TCGA’s cohort, while the blue line indicates a higher mutation frequency in the corresponding pathway favors in our cohort.

**c.** Significant enrichment of pathway mutations in breast cancer patients at different stages.

**d.** Significant enrichment of pathway mutations in breast cancer patients of different ages.

**e.** Distribution of VAFs in mutations in oncogenic signaling pathways.

**f-i.** Significant mutual exclusivity (blue) and co-occurrence (red) of gene mutations among pathways in different subtypes of breast cancer (luminal A, **f**; luminal B/HER2+, **g**; HER2-positive, **h**; triple negative, **i**). Spectrum bar: log10 (odds ratio (OR)); the color intensity represents the scale of the value.

Source data for a-b are provided as a source data file.

**Supplementary Figure 9**
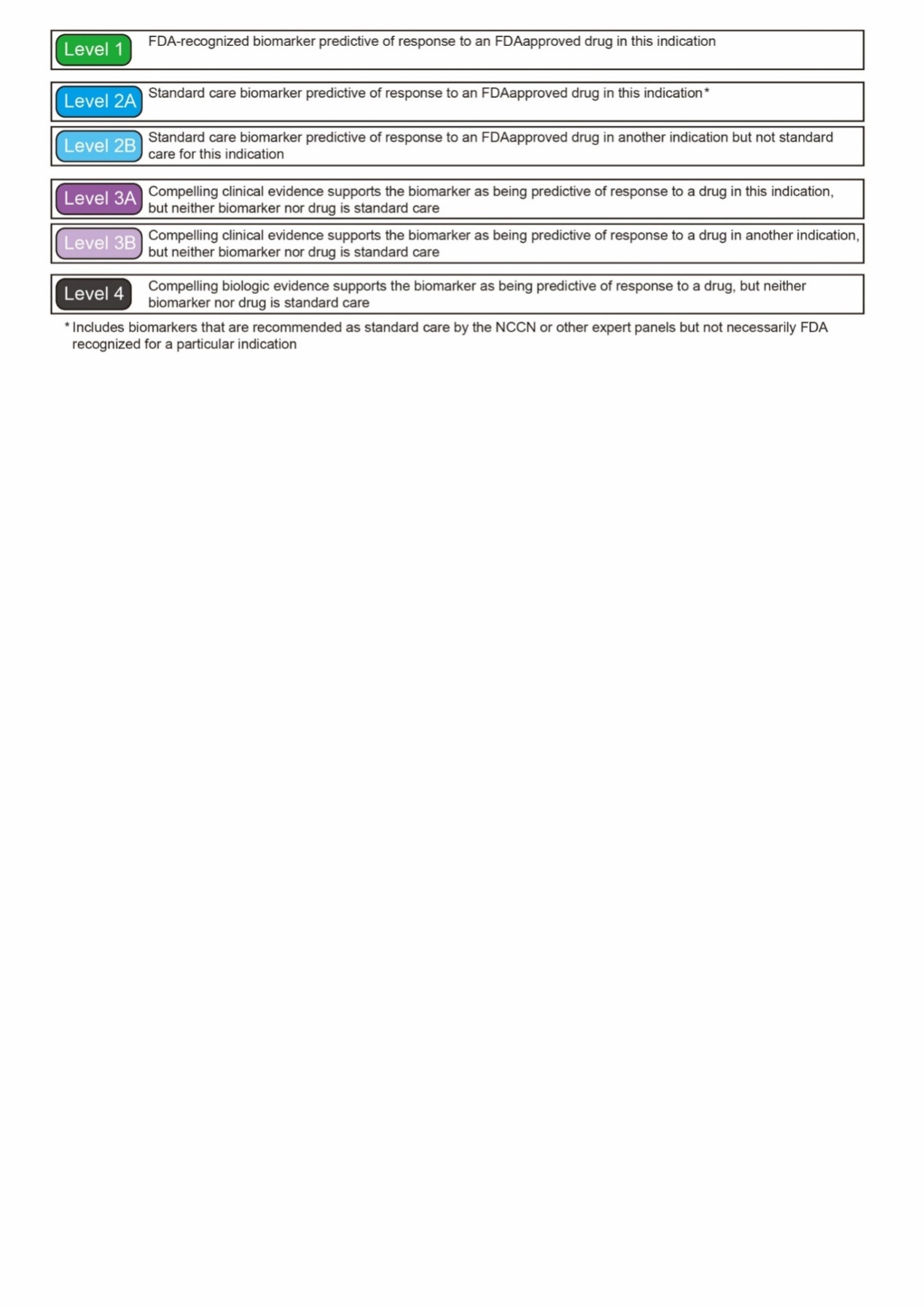


**Supplementary Fig.9丨Levels of evidence in the Fudan Breast Cancer Precision Medicine Knowledge Base.**

**Supplementary Figure 10**
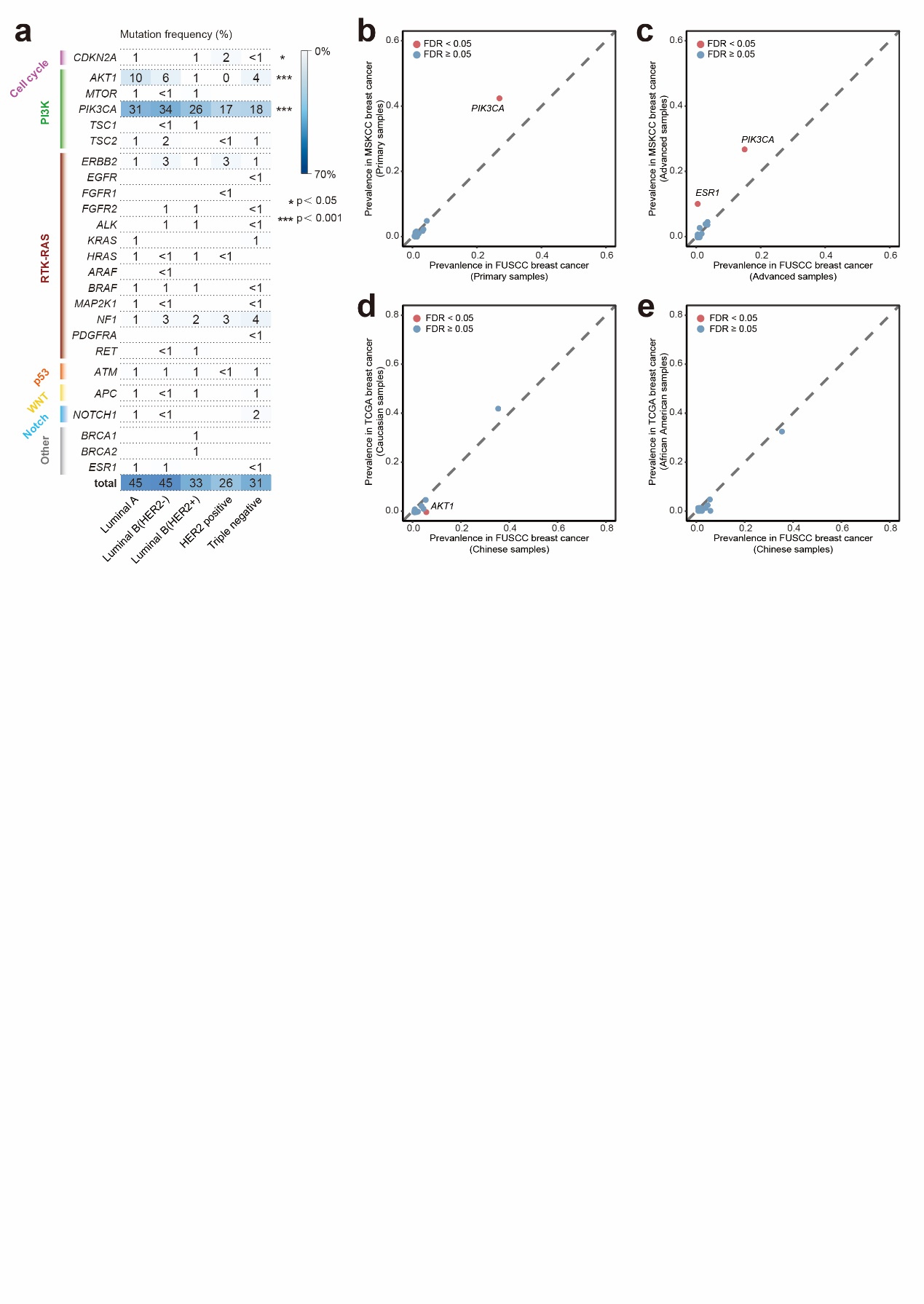


**Supplementary Fig.10丨 Actionable and oncogenic alterations revealed by clinical sequencing.**

**a.** Frequencies of actionable mutations per gene across molecular subtypes of breast cancer. Genes are grouped by pathway. The last row of each pathway indicates the overall actionable mutation frequency in the pathway in each breast cancer subtype. The asterisks indicate a statistically significant association with the subtype (Chi-square test, two-sided)

**b and c.** Scatter plots of the prevalence of actionable mutation spots in primary (**b**) and advanced (**c**) breast cancer samples from FUSCC (x-axis) and the MSKCC dataset (y-axis), according to the oncoKB criteria.

**d and e.** Scatter plots of the prevalence of mutated genes in breast cancer samples from the FUSCC (x-axis) and the Caucasian (**d**) and African American (**e**) samples in the TCGA dataset (y-axis).

Source data for a are provided as a source data file.

**Supplementary Figure 11**


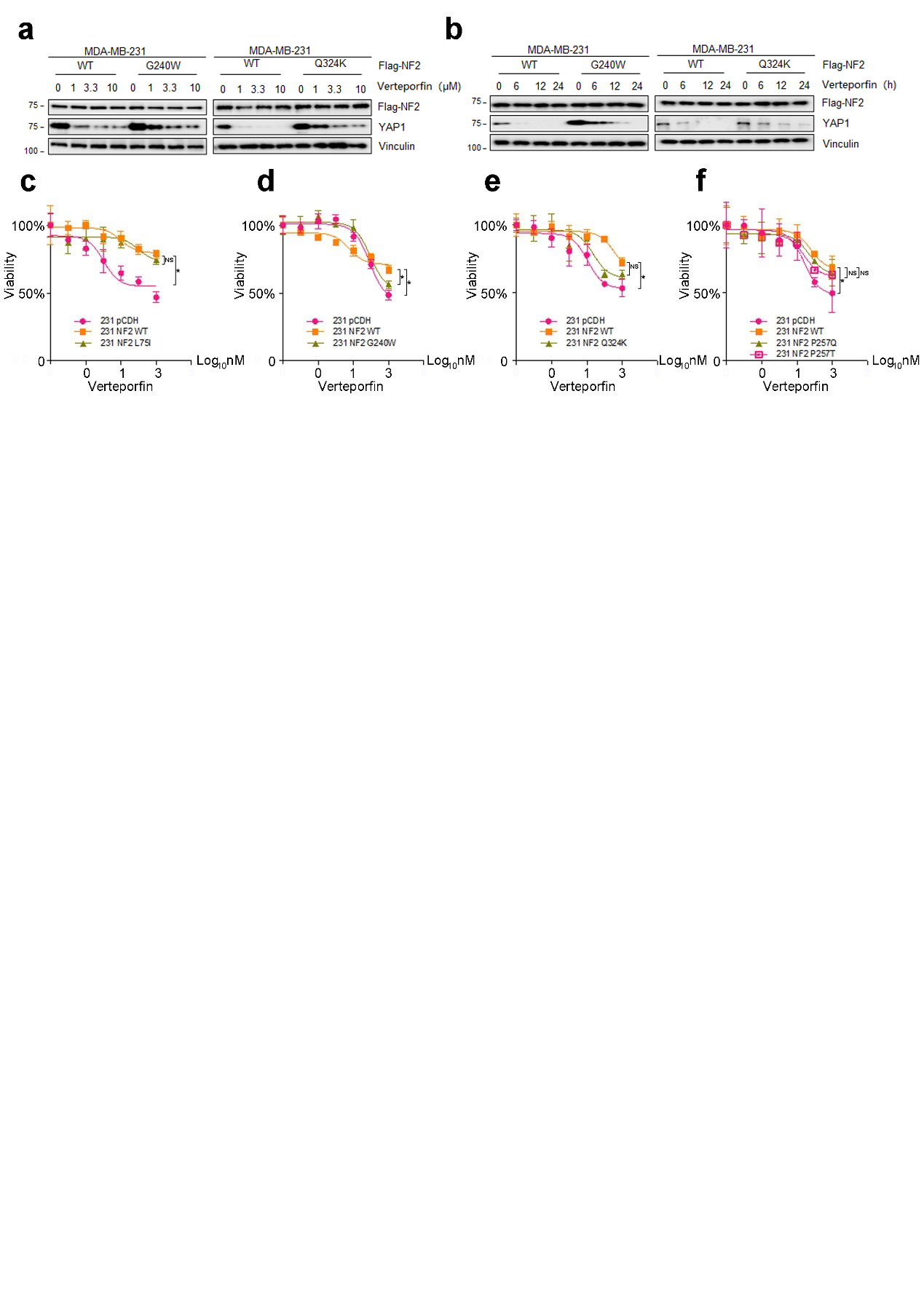
**Supplementary Fig. 11丨 *NF2* mutations promote sensitivity to a YAP inhibitor.**

**a.** MDA-MB-231 cells stably expressing Flag-*NF2*-WT or Flag-*NF2*-G240W/Q324K were treated with increasing doses of Verteporfin.

**b.** MDA-MB-231 cells stably expressing Flag-*NF2*-WT or Flag-*NF2*-G240W/Q324K were treated with 1 μM Verteporfin for 0, 6, 12 and 24 h. All western blots experiments are repeated three times.

**c-f.** Relative percentage of cell viability (%) of *NF2*-WT and *NF2*-mutated (G240W, **c**; Q324K, **d**; L75I, **e**; P257Q/T, **f**) MDA-MB-231 cells treated with Verteporfin. The half-maximal inhibitory concentration (IC50) values were calculated based on the day 3 data of various doses of drug treatment. The assays were performed with 5 replicates in 3 independent experiments; representative results are shown. These data represent the mean values, with error bars indicating the SEM (*, P < 0.05).

| **Supplementary Table 1 丨The comparison of patient characteristics between FUSCC and TCGA/MSKCC cohorts.** | | | | | | | |  |
| --- | --- | --- | --- | --- | --- | --- | --- | --- |
| **Variables** | **FUSCC (n=1134)** | | **TCGA (n=982)** | | **MSKCC (n = 1756)** | | ***P1*** | ***P2*** |
|  | **No.** | **(%)** | **No.** | **(%)** | **No.** | **(%)** |  |  |
| Age |  |  |  |  |  |  | ＜0.001 | 0.246 |
| ≤50 years | 493 | 43% | 298 | 30% | 802 | 46% |  |  |
| >50 years | 641 | 57% | 684 | 70% | 954 | 54% |  |  |
| Sex |  |  |  |  |  |  | 0.001 | 0.026 |
| Female | 1134 | 100% | 971 | 99% | 1746 | 99% |  |  |
| Male | 0 | 0% | 11 | 1% | 10 | 1% |  |  |
| LN status |  |  |  |  |  |  | 0.137 | ＜0.001 |
| Positive | 647 | 57% | 510 | 52% | 755 | 43% |  |  |
| Negative | 464 | 41% | 456 | 46% | 745 | 42% |  |  |
| Unknown | 23 | 2% | 16 | 2% | 246 | 14% |  |  |
| TNM stage |  |  |  |  |  |  | ＜0.001 | 0.040 |
| I–II | 661 | 58% | 715 | 73% | 1029 | 59% |  |  |
| III–IV | 453 | 40% | 244 | 25% | 714 | 41% |  |  |
| Unknown | 20 | 2% | 23 | 2% | 13 | 1% |  |  |
| Pathological type | |  |  |  |  |  | ＜0.001 | ＜0.001 |
| IDC | 658 | 58% | 728 | 74% | 1339 | 76% |  |  |
| ILC | 12 | 1% | 165 | 17% | 275 | 16% |  |  |
| Others | 57 | 5% | 89 | 9% | 113 | 6% |  |  |
| Unknown | 407 | 36% | 0 | 0% | 29 | 2% |  |  |
| ER status |  |  |  |  |  |  | ＜0.001 | ＜0.001 |
| Positive |  |  |  |  |  |  |  |  |
| Negative | 414 | 37% | 210 | 21% | 472 | 27% |  |  |
| Unknown | 0 | 0% | 45 | 5% | 40 | 2% |  |  |
| PR status |  |  |  |  |  |  | ＜0.001 | ＜0.001 |
| Positive | 594 | 52% | 631 | 64% | 1244 | 71% |  |  |
| Negative | 540 | 48% | 305 | 31% | 472 | 27% |  |  |
| Unknown | 0 | 0% | 45 | 5% | 40 | 2% |  |  |
| HER2 status | |  |  |  |  |  | ＜0.001 | ＜0.001 |
| Positive | 516 | 46% | 168 | 17% | 190 | 11% |  |  |
| Negative | 603 | 53% | 645 | 66% | 1462 | 83% |  |  |
| Unknown | 15 | 1% | 169 | 17% | 104 | 6% |  |  |
| Histologic grade | |  |  |  |  |  | NA | ＜0.001 |
| I | 11 | 1% | 0 | 0% | 90 | 5% |  |  |
| II | 310 | 27% | 0 | 0% | 422 | 24% |  |  |
| III | 322 | 28% | 0 | 0% | 1071 | 61% |  |  |
| Unknown | 491 | 43% | 982 | 100% | 173 | 10% |  |  |

Associations were evaluated by two-sided Chi-square test. *P1*, FUSCC vs TCGA; *P2*, FUSCC vs MSKCC.

|  | | | | | | | | |
| --- | --- | --- | --- | --- | --- | --- | --- | --- |
| **Supplementary Table 2丨The comparison of sample sites of advanced patients between FUSCC and MSKCC cohorts.** | | | | | | | |  |
| **Metastatic sites** | **FUSCC (n = 109)** | | **MSKCC (n = 905)** | | ***P*** |  |  |  |
|  | **No.** | **(%)** | **No.** | **(%)** |  |  |  |  |
| Liver | 9 | 8.3% | 206 | 22.8% | ＜0.001 |  |  |  |
| Bone | 0 | 0.0% | 128 | 14.1% |  |  |  |  |
| Lymph Node | 9 | 8.3% | 126 | 13.9% |  |  |  |  |
| Chest Wall | 8 | 7.3% | 79 | 8.7% |  |  |  |  |
| Lung | 5 | 4.6% | 64 | 7.1% |  |  |  |  |
| Pleura | 0 | 0.0% | 39 | 4.3% |  |  |  |  |
| Brain | 0 | 0.0% | 33 | 3.6% |  |  |  |  |
| Breast | 78 | 71.6% | 29 | 3.2% |  |  |  |  |
| Skin | 0 | 0.0% | 25 | 2.8% |  |  |  |  |
| Ovary | 0 | 0.0% | 25 | 2.8% |  |  |  |  |
| Soft Tissue | 0 | 0.0% | 24 | 2.7% |  |  |  |  |
| Peritoneum | 0 | 0.0% | 8 | 0.9% |  |  |  |  |
| Epidural Mass | 0 | 0.0% | 7 | 0.8% |  |  |  |  |
| Bowel | 0 | 0.0% | 7 | 0.8% |  |  |  |  |
| Bladder/Ureter | 0 | 0.0% | 5 | 0.6% |  |  |  |  |
| Stomach | 0 | 0.0% | 4 | 0.4% |  |  |  |  |
| Orbit | 0 | 0.0% | 4 | 0.4% |  |  |  |  |
| Uterus | 0 | 0.0% | 2 | 0.2% |  |  |  |  |
| Pericardium | 0 | 0.0% | 2 | 0.2% |  |  |  |  |
| Trachea | 0 | 0.0% | 1 | 0.1% |  |  |  |  |
| Spleen | 0 | 0.0% | 1 | 0.1% |  |  |  |  |
| Parotid | 0 | 0.0% | 1 | 0.1% |  |  |  |  |
| Esophegus | 0 | 0.0% | 1 | 0.1% |  |  |  |  |
| Multiple sites | 0 | 0.0% | 84 | 9.3% |  |  |  |  |
| Associations were evaluated by two-sided Chi-square test. | | | | | |  |  |  |

| **Supplementary table 3 \| Common genes in different panels.** | | | |
| --- | --- | --- | --- |
| **Genes** | **FUSCC-BC** | **MSK-IMPACT** | **Foundation One** |
| *ABL1* | + | + | + |
| *AKT1* | + | + | + |
| *AKT2* | + | + | + |
| *AKT3* | + | + | + |
| *ALK* | + | + | + |
| *ANKRD11* | + | + | - |
| *APC* | + | + | + |
| *ARAF* | + | + | + |
| *ARID1A* | + | + | + |
| *ARID1B* | + | + | - |
| *ARID2* | + | + | - |
| *ASXL1* | + | + | + |
| *ATM* | + | + | + |
| *ATR* | + | + | + |
| *ATRX* | + | + | + |
| *AURKA* | + | + | + |
| *AURKB* | + | + | + |
| *AXIN1* | + | + | + |
| *AXL* | + | + | + |
| *BCL2* | + | + | + |
| *BCL2L2* | + | - | + |
| *BCOR* | + | + | + |
| *BCR* | + | - | + |
| *BIRC3* | + | + | - |
| *BRAF* | + | + | + |
| *BRCA1* | + | + | + |
| *BRCA2* | + | + | + |
| *BTK* | + | + | + |
| *CASP8* | + | + | + |
| *CBFB* | + | + | + |
| *CBL* | + | + | + |
| *CCND1* | + | + | + |
| *CCND2* | + | + | + |
| *CCND3* | + | + | + |
| *CCNE1* | + | + | + |
| *CD274* | + | + | + |
| *CDH1* | + | + | + |
| *CDK4* | + | + | + |
| *CDK6* | + | + | + |
| *CDKN1B* | + | + | + |
| *CDKN2A* | + | + | + |
| *CDKN2B* | + | + | + |
| *CHEK1* | + | + | + |
| *CHEK2* | + | + | + |
| *CIC* | + | + | + |
| *CREBBP* | + | + | + |
| *CSF1R* | + | + | + |
| *CSF3R* | + | + | + |
| *CTCF* | + | + | + |
| *CUL4A* | + | - | + |
| *DNMT1* | + | + | - |
| *DNMT3A* | + | + | + |
| *DNMT3B* | + | + | - |
| *EGFR* | + | + | + |
| *EPAS1* | + | + | - |
| *EPHA3* | + | + | + |
| *EPHB1* | + | + | + |
| *EPHB4* | + | - | + |
| *ERBB2* | + | + | + |
| *ERBB3* | + | + | + |
| *ERBB4* | + | + | + |
| *ERCC4* | + | + | + |
| *ESR1* | + | + | + |
| *FBXW7* | + | + | + |
| *FGFR1* | + | + | + |
| *FGFR2* | + | + | + |
| *FGFR3* | + | + | + |
| *FGFR4* | + | + | + |
| *FH* | + | + | + |
| *FLT1* | + | + | + |
| *FLT3* | + | + | + |
| *FLT4* | + | + | - |
| *FOXA1* | + | + | - |
| *FOXP1* | + | + | - |
| *FYN* | + | + | - |
| *GATA3* | + | + | + |
| *GNAS* | + | + | + |
| *GPS2* | + | + | - |
| *GRIN2A* | + | + | - |
| *GSK3B* | + | + | + |
| *HDAC1* | + | - | + |
| *HIST1H1C* | + | + | - |
| *HIST1H3B* | + | + | - |
| *HRAS* | + | + | + |
| *IGF1* | + | + | - |
| *IGF1R* | + | + | + |
| *IL7R* | + | + | - |
| *INSR* | + | + | - |
| *IRF4* | + | + | + |
| *IRS1* | + | + | - |
| *IRS2* | + | + | + |
| *JAK1* | + | + | + |
| *JAK2* | + | + | + |
| *JAK3* | + | + | + |
| *JUN* | + | + | + |
| *KDM5A* | + | + | + |
| *KDM6A* | + | + | + |
| *KDR* | + | + | + |
| *KIT* | + | + | + |
| *KMT2B* | + | + | - |
| *KMT2C* | + | + | - |
| *KMT2D* | + | + | + |
| *KRAS* | + | + | + |
| *LYN* | + | + | + |
| *MAP2K1* | + | + | + |
| *MAP2K2* | + | + | + |
| *MAP2K4* | + | + | + |
| *MAP3K1* | + | + | + |
| *MAP3K13* | + | + | + |
| *MAPK1* | + | + | + |
| *MAPK3* | + | + | - |
| *MCL1* | + | + | + |
| *MDM2* | + | + | + |
| *MDM4* | + | + | + |
| *MEN1* | + | + | + |
| *MERTK* | + | - | + |
| *MET* | + | + | + |
| *MLH1* | + | + | + |
| *MSH2* | + | + | + |
| *MST1R* | + | + | + |
| *MTAP* | + | - | + |
| *MTOR* | + | + | + |
| *MYB* | + | - | + |
| *MYC* | + | + | + |
| *NCOR1* | + | + | - |
| *NF1* | + | + | + |
| *NF2* | + | + | + |
| *NFKBIA* | + | + | + |
| *NOTCH1* | + | + | + |
| *NOTCH2* | + | + | + |
| *NOTCH3* | + | + | + |
| *NRAS* | + | + | + |
| *NTRK1* | + | + | + |
| *NTRK2* | + | + | + |
| *PAK1* | + | + | - |
| *PALB2* | + | + | + |
| *PARP1* | + | + | + |
| *PARP2* | + | - | + |
| *PBRM1* | + | + | + |
| *PDGFRA* | + | + | + |
| *PDGFRB* | + | + | + |
| *PDPK1* | + | + | - |
| *PGR* | + | + | - |
| *PIK3C3* | + | + | - |
| *PIK3CA* | + | + | + |
| *PIK3CB* | + | + | + |
| *PIK3CD* | + | + | - |
| *PIK3CG* | + | + | - |
| *PIK3R1* | + | + | + |
| *PIK3R2* | + | + | - |
| *PIM1* | + | + | + |
| *PLK2* | + | + | - |
| *PMS2* | + | + | + |
| *PPARG* | + | + | + |
| *PRDM1* | + | + | + |
| *PREX2* | + | + | - |
| *PTEN* | + | + | + |
| *PTPRD* | + | + | - |
| *RAC1* | + | + | + |
| *RAC2* | + | + | - |
| *RAD51* | + | + | + |
| *RAF1* | + | + | + |
| *RB1* | + | + | + |
| *REL* | + | + | + |
| *RET* | + | + | + |
| *RHOA* | + | + | - |
| *RICTOR* | + | + | + |
| *RPTOR* | + | + | + |
| *RUNX1* | + | + | - |
| *SETD2* | + | + | + |
| *SF3B1* | + | + | + |
| *SGK1* | + | - | + |
| *SMAD4* | + | + | + |
| *SMARCA4* | + | + | + |
| *SMO* | + | + | + |
| *SMYD3* | + | + | - |
| *SOX9* | + | + | + |
| *SPEN* | + | + | + |
| *SRC* | + | + | + |
| *STAG2* | + | + | + |
| *STAT5A* | + | + | - |
| *STAT5B* | + | + | - |
| *STK11* | + | + | + |
| *SYK* | + | + | + |
| *TBX3* | + | + | + |
| *TEK* | + | + | + |
| *TERT* | + | + | + |
| *TET2* | + | + | + |
| *TGFBR1* | + | + | - |
| *TOP1* | + | + | - |
| *TP53* | + | + | + |
| *TSC1* | + | + | + |
| *TSC2* | + | + | + |
| *TYRO3* | + | - | + |
| *VHL* | + | + | + |
| *WHSC1L1* | + | + | + |
| *YES1* | + | + | - |
| *ALOX12B* | - | + | + |
| *AMER1* | - | + | + |
| *AR* | - | + | + |
| *BAP1* | - | + | + |
| *BARD1* | - | + | + |
| *BCL2L1* | - | + | + |
| *BCL6* | - | + | + |
| *BRD4* | - | + | + |
| *BRIP1* | - | + | + |
| *CALR* | - | + | + |
| *CARD11* | - | + | + |
| *CD79A* | - | + | + |
| *CD79B* | - | + | + |
| *CDC73* | - | + | + |
| *CDK12* | - | + | + |
| *CDK8* | - | + | + |
| *CDKN1A* | - | + | + |
| *CDKN2C* | - | + | + |
| *CEBPA* | - | + | + |
| *CRKL* | - | + | + |
| *CTNNB1* | - | + | + |
| *CUL3* | - | + | + |
| *CXCR4* | - | + | + |
| *DAXX* | - | + | + |
| *DDR2* | - | + | + |
| *DIS3* | - | + | + |
| *DOT1L* | - | + | + |
| *EED* | - | + | + |
| *EP300* | - | + | + |
| *ERG* | - | + | + |
| *ERRFI1* | - | + | + |
| *ETV6* | - | + | + |
| *EZH2* | - | + | + |
| *FAM46C* | - | + | + |
| *FANCA* | - | + | + |
| *FANCC* | - | + | + |
| *FGF19* | - | + | + |
| *FGF3* | - | + | + |
| *FGF4* | - | + | + |
| *FLCN* | - | + | + |
| *FOXL2* | - | + | + |
| *FUBP1* | - | + | + |
| *GNA11* | - | + | + |
| *GNAQ* | - | + | + |
| *H3F3A* | - | + | + |
| *HGF* | - | + | + |
| *HNF1A* | - | + | + |
| *ID3* | - | + | + |
| *IDH1* | - | + | + |
| *IDH2* | - | + | + |
| *IKBKE* | - | + | + |
| *IKZF1* | - | + | + |
| *INPP4B* | - | + | + |
| *KDM5C* | - | + | + |
| *KEAP1* | - | + | + |
| *KMT2A* | - | + | + |
| *MED12* | - | + | + |
| *MEF2B* | - | + | + |
| *MITF* | - | + | + |
| *MPL* | - | + | + |
| *MRE11A* | - | + | + |
| *MSH3* | - | + | + |
| *MSH6* | - | + | + |
| *MUTYH* | - | + | + |
| *MYCN* | - | + | + |
| *MYD88* | - | + | + |
| *NBN* | - | + | + |
| *NFE2L2* | - | + | + |
| *NKX2-1* | - | + | + |
| *NPM1* | - | + | + |
| *NTRK3* | - | + | + |
| *PARK2* | - | + | + |
| *PAX5* | - | + | + |
| *PDCD1* | - | + | + |
| *PDCD1LG2* | - | + | + |
| *PIK3C2G* | - | + | + |
| *POLD1* | - | + | + |
| *POLE* | - | + | + |
| *PPP2R1A* | - | + | + |
| *PRKAR1A* | - | + | + |
| *PRKCI* | - | + | + |
| *PTCH1* | - | + | + |
| *PTPN11* | - | + | + |
| *RAD21* | - | + | + |
| *RAD51B* | - | + | + |
| *RAD51C* | - | + | + |
| *RAD51D* | - | + | + |
| *RAD52* | - | + | + |
| *RAD54L* | - | + | + |
| *RARA* | - | + | + |
| *RBM10* | - | + | + |
| *RNF43* | - | + | + |
| *ROS1* | - | + | + |
| *SDHA* | - | + | + |
| *SDHB* | - | + | + |
| *SDHC* | - | + | + |
| *SDHD* | - | + | + |
| *SMAD2* | - | + | + |
| *SMARCB1* | - | + | + |
| *SOCS1* | - | + | + |
| *SOX2* | - | + | + |
| *SPOP* | - | + | + |
| *STAT3* | - | + | + |
| *SUFU* | - | + | + |
| *TGFBR2* | - | + | + |
| *TMPRSS2* | - | + | + |
| *TNFAIP3* | - | + | + |
| *TNFRSF14* | - | + | + |
| *U2AF1* | - | + | + |
| *VEGFA* | - | + | + |
| *WHSC1* | - | + | + |
| *WT1* | - | + | + |
| *XPO1* | - | + | + |
| *XRCC2* | - | + | + |

| **Supplementary Table 4. Groupwise comparison of mutational frequency by molecular subtype.** | | | | | | | | |
| --- | --- | --- | --- | --- | --- | --- | --- | --- |
| **Gene** | **Group 1** | **Group 2** | **Group 1** | | **Group 2** | | ***P*** | **FDR** |
|  |  |  | **Total** | **Mutated** | **Total** | **Mutated** |  |  |
| *TP53* | Triple negative | Rest | 206 | 164 | 928 | 441 | ＜0.001 | ＜0.001 |
| *TP53* | HER2 | Rest | 206 | 153 | 928 | 452 | ＜0.001 | ＜0.001 |
| *PIK3CA* | Luminal B (HER2-) | Rest | 393 | 102 | 741 | 117 | ＜0.001 | 0.012 |
| *PTEN* | Triple negative | Rest | 206 | 21 | 928 | 28 | ＜0.001 | 0.012 |
| *AKT1* | Luminal A | Rest | 139 | 17 | 995 | 37 | ＜0.001 | 0.029 |
| *FAM47C* | Triple negative | Rest | 206 | 6 | 928 | 2 | ＜0.001 | 0.155 |
| *GATA3* | Luminal B (HER2-) | Rest | 393 | 52 | 741 | 54 | ＜0.001 | 0.176 |
| *CBFB* | Luminal A | Rest | 139 | 10 | 995 | 18 | 0.001 | 0.176 |
| *KDM6A* | Triple negative | Rest | 206 | 9 | 928 | 8 | 0.001 | 0.176 |
| *XDH* | Luminal B (HER2-) | Rest | 393 | 9 | 741 | 2 | 0.002 | 0.245 |

Associations were evaluated by two-sided Fisher’s exact test.

| **Supplementary table 5 \| Summary of genomic characteristics and targets of different molecular subtypes in Chinese breast cancer.** | | | | | |
| --- | --- | --- | --- | --- | --- |
| Subtype | Luminal A | Luminal B (HER2-) | Luminal B (HER2+) | HER2 positive | Triple negative |
| Mutation profile | *CDKN1B* (2%)↑ | *CDKN1B* (0%)↓ | *TSC1* (3%)↑ | *ATK1* (1%)↓ | *CDKN1B* (0%)↓ |
|  | *AKT1* (12%)↑ | *PIK3CA* (38%)↑ | *NTRK2* (3%)↑ | *PIK3CA* (24%)↓ | *PTEN* (10%)↑ |
|  | *NTRK2* (0%)↓ | *NTRK2* (0%)↓ | *ATM* (5%)↑ | *PTEN* (0%)↓ | *TP53* (80%)↑ |
|  |  |  |  | *TSC1* (0%)↓ |  |
|  |  |  |  | *ATM* (1%)↓ |  |
| Pathway profile | P53 (22%)↓ | PI3K (41%)↑ | Hippo (0%)↓ | PI3K (18%)↓ | Hippo (4%)↑ |
|  |  |  |  |  | P53 (22%)↓ |
| Comparing with foreign mutational profile | Main difference | | No significant difference | | |
| Co-occuring pathways | Notch & RTK-RAS | Cell Cycle & RTK-RAS | Cell Cycle & WNT | Notch & RTK-RAS | PI3K & WNT |
|  | WNT & RTK-RAS | Hippo & WNT | Notch & WNT |  | PI3K & RTK-RAS |
|  |  | Hippo & TGF-Beta | Notch & RTK-RAS |  | WNT & RTK-RAS |
|  |  | Notch & RTK-RAS | WNT & RTK-RAS |  |  |
|  |  | WNT & TGF-Beta |  |  |  |
| Mutually exclusive pathways |  | PI3K & RTK-RAS | WNT & P53 | Cell Cycle & P53 | PI3K & P53 |
|  |  | PI3K & P53 | RTK-RAS & P53 | Notch & P53 | RTK-RAS & P53 |
|  |  |  |  | PI3K & P53 |  |
| Potential actionable strategies | PI3K inhibitors | PI3K inhibitors | Cell Cycle inhibitors + WNT inhibitors | Notch inhibitors + RTK-RAS inhibitors | PI3K inhibitors |
|  | Notch inhibitors + RTK-RAS inhibitors | Cell Cycle inhibitors + RTK-RAS inhibitors | Notch inhibitors + WNT inhibitors |  | Hippo inhibitors |
|  |  | Hippo inhibitors + WNT inhibitors | Notch inhibitors + RTK-RAS inhibitors |  | PI3K inhibitors + WNT inhibitors |
|  |  | Notch inhibitors + RTK-RAS inhibitors | WNT inhibitors + RTK-RAS inhibitors |  | WNT inhibitors + RTK-RAS inhibitors |

| **Supplementary table 6 \| Primers used for molecular cloning of expression vectors.** | | |
| --- | --- | --- |
| Plamids | Primers | Sequences |
| Flag-NF2-L75I | Forward | GACACAGTGGCCTGGATCAAAATAGACAAG |
|  | Reverse | CTTGTCTATTTTGATCCAGGCCACTGTGTC |
| Flag-NF2-G240W | Forward | GAGTGGATGCCCTGTGGCTTCACATTTATG |
|  | Reverse | CATAAATGTGAAGCCACAGGGCATCCACTC |
| Flag-NF2-Q324K | Forward | CAGATGAAAGCCAAGGCCAGGGAGG |
|  | Reverse | CCTCCCTGGCCTTGGCTTTCATCTG |
| Flag-NF2-P257T | Forward | AACAGACTGACCCCCAAGATCTCCTTCACGTGGAATGAAATC |
|  | Reverse | GTACGAGATGTTTCGGATTTCATTCCACGTGAAGGAGATCTT |
| Flag-NF2-P257Q | Forward | AACAGACTGACCCCCAAGATCTCCTTCCAGTGGAATGAAATC |
|  | Reverse | GTACGAGATGTTTCGGATTTCATTCCACTGGAAGGAGATCTT |

| **Supplementary table 7 \| Detailed information of antibodies in this study.** | | | | |
| --- | --- | --- | --- | --- |
| Antibodies | Vendors | Cat# | Species | Working concentration |
| NF2 | Abcam | ab109244 | Rabbit | 1:1000 |
| FGFR2 | Abcam | ab109372 | Rabbit | 1:1000 |
| JNK | Abcam | ab208035 | Rabbit | 1:1000 |
| p-JNK(phospho T183+T183+T221) | Abcam | ab124956 | Rabbit | 1:1000 |
| p38 | Abcam | ab170099 | Rabbit | 1:1000 |
| p-p38(phospho T180) | Abcam | ab178867 | Rabbit | 1:1000 |
| YAP1 | Abcam | ab52771 | Rabbit | 1:1000 |
| p-YAP1(phospho S127) | Abcam | ab76252 | Rabbit | 1:1000 |
| p-Rb(phospho S780) | Abcam | ab173289 | Rabbit | 1:1000 |
| cyclinD1 | Abcam | ab134175 | Rabbit | 1:1000 |
| Vinculin | Sigma | V9131 | Mouse | 1:3000 |
