# Supplementary material for: Characterization of the genomic landscape and actionable mutations in Chinese breast cancers by clinical sequencing
Source: Nat Commun. 2020 Nov 10;11:5679. doi: 10.1038/s41467-020-19342-3 (PMC7656255; doi:10.1038/s41467-020-19342-3)
Supplement: Supplementary file 3 — Description of Additional Supplementary Files [file 41467_2020_19342_MOESM3_ESM.pdf]

Description of Additional Supplementary Files

Title: Supplementary Data 1

Description: Sequencing gene content of the prospective cohort.

Title: Supplementary Data 2

Description: Basic information of breast cancer patients in our study.

Title: Supplementary Data 3

Description: Fudan Breast Cancer Precision Medicine Knowledge Base (FBC PreMedKB).

Title: Supplementary Data 4

Description: Patient-based oncogenic and actionable mutations and corresponding drugs in our study.
