# Supplementary material for: Characterization of the genomic landscape and actionable mutations in Chinese breast cancers by clinical sequencing
Source: Nat Commun. 2020 Nov 10;11:5679. doi: 10.1038/s41467-020-19342-3 (PMC7656255; doi:10.1038/s41467-020-19342-3)
Supplement: Supplementary file 8 — Reporting Summary [file 41467_2020_19342_MOESM8_ESM.pdf]

nature research

Corresponding author(s): Zhi-Ming Shao; Wei Huang; Xin Hu

Last updated by author(s): Sep 20, 2020

Reporting Summary

Nature Research wishes to improve the reproducibility of the work that we publish. This form provides structure for consistency and transparency in reporting. For further information on Nature Research policies, see Authors & Referees and the Editorial Policy Checklist .

Statistics

For all statistical analyses, confirm that the following items are present in the figure legend, table legend, main text, or Methods section.

n/a	Confirmed	
☐	☒	The exact sample size (n) for each experimental group/condition, given as a discrete number and unit of measurement
☐	☒	A statement on whether measurements were taken from distinct samples or whether the same sample was measured repeatedly
☐	☒	The statistical test(s) used AND whether they are one- or two-sided<br>*Only common tests should be described solely by name; describe more complex techniques in the Methods section.*
☐	☒	A description of all covariates tested
☐	☒	A description of any assumptions or corrections, such as tests of normality and adjustment for multiple comparisons
☐	☒	A full description of the statistical parameters including central tendency (e.g. means) or other basic estimates (e.g. regression coefficient) AND variation (e.g. standard deviation) or associated estimates of uncertainty (e.g. confidence intervals)
☐	☒	For null hypothesis testing, the test statistic (e.g. F, t, r) with confidence intervals, effect sizes, degrees of freedom and P value noted<br>*Give P values as exact values whenever suitable.*
☒	☐	For Bayesian analysis, information on the choice of priors and Markov chain Monte Carlo settings
☒	☐	For hierarchical and complex designs, identification of the appropriate level for tests and full reporting of outcomes
☒	☐	Estimates of effect sizes (e.g. Cohen's d, Pearson's r), indicating how they were calculated

Our web collection on statistics for biologists contains articles on many of the points above.

Software and code

Policy information about availability of computer code

Data collection	Data was collected using Illumina Real Time Analysis (RTA) 3 and assembled to fastq files using Illumina Bcl2Fastq2 v2.19.
Data analysis	High-quality reads were mapped to the hg19 version of the human reference genome (GRCh37) using the BWA (version 0.7.17-r1188) aligner with the BWA-MEM algorithm and default parameters. The Genome Analysis Toolkit (GATK) was used to locally realign the BAM files at intervals with mismatched InDels and recalibrate the base quality scores of the reads in the BAM files. Germline variants from the blood BAM file were identified using GATK (version 4.0.11.0) HaplotypeCaller. CharGer was used to classify the germline variants (version 0.5.4). Somatic mutations were called from the tissue and blood BAM files using GATK (version 4.0.12.0) Mutect2 with the default parameters. The sequencing quality statistics were obtained using SAMtools (version 2.6.2) and GATK (version 4.0.12.0) . The VCF files were annotated using ANNOVAR (version 2015-06-17). The DNA copy number variations were determined through the FACETS algorithm (version 0.16.0). All statistical analyses were performed using R package (version 3.4.2).<br>All codes and scripts are available on https://github.com/ninnywolf/FUSCC-PCMC-BRCA-target-sequencing.git.<br>The IC50 was calculated using GraphPad Prism (version 7.0).

For manuscripts utilizing custom algorithms or software that are central to the research but not yet described in published literature, software must be made available to editors/reviewers. We strongly encourage code deposition in a community repository (e.g. GitHub). See the Nature Research guidelines for submitting code & software for further information.

Data

Policy information about availability of data

All manuscripts must include a data availability statement. This statement should provide the following information, where applicable:

- Accession codes, unique identifiers, or web links for publicly available datasets
- A list of figures that have associated raw data
- A description of any restrictions on data availability

The authors declare that the DNA sequencing dataset in our study is deposited in the publicly accessible database from the Chinese Academy of Sciences. All data

can be viewed in NODE (http://www.biosino.org/node) by pasting the accession number (OEP001027) into the text search box or through the following URL: http://www.biosino.org/node/project/detail/OEP001027. The sequence data have also been deposited in the NCBI Sequence Read Archive (SRA) database under the accession code SRP282257, SRP282270 and SRP282290. All data can be viewed in the SRA website (https://www.ncbi.nlm.nih.gov/sra) by pasting the accession number (SRP282257, SRP282270 and SRP282290) into the text search box or through the following hyperlinks: https://trace.ncbi.nlm.nih.gov/Traces/study/?acc=SRP282257, https://trace.ncbi.nlm.nih.gov/Traces/study/?acc=SRP282270, and https://trace.ncbi.nlm.nih.gov/Traces/study/?acc=SRP282290.
The public data that support the findings of this study are available from cBioPortal (http://download.cbioportal.org/breast_msk_2018.tar.gz , http://download.cbioportal.org/brca_tcga.tar.gz) and from Fudan Data Portal (http://data.3steps.cn/cdataportal/study/summary?id=FUSCC_BRCA_panel_1000).
All the other data supporting the findings of this study are available within the article and its supplementary information files and from the corresponding author upon reasonable request. A reporting summary for this article is available as a Supplementary Information file.

Field-specific reporting

Please select the one below that is the best fit for your research. If you are not sure, read the appropriate sections before making your selection.

[x] Life sciences [ ] Behavioural & social sciences [ ] Ecological, evolutionary & environmental sciences

For a reference copy of the document with all sections, see nature.com/documents/nr-reporting-summary-flat.pdf

Life sciences study design

All studies must disclose on these points even when the disclosure is negative.

Sample size

We recruited 1,134 patients who were treated at the Department of Breast Surgery at Fudan University Shanghai Cancer Center from April 1, 2018, to April 1, 2019 to establish the largest sequencing database of Chinese breast cancer. The sample size in our study was comparable with the sample size of breast cancers in TCGA.

Data exclusions

The exclusion criteria were pre-established that samples without sufficient tissue for sequencing by core needle biopsy or data which failed sequencing quality control were excluded before enrollment.

Replication

The assays were performed with 5 replicates in 3 independent experiments.

Randomization

No randomization was needed in the clinical sequencing study for all participants were enrolled and studied in the common procedure.

Blinding

No blinding was needed in the clinical sequencing study for all participants were enrolled and studied in the common procedure.

Reporting for specific materials, systems and methods

We require information from authors about some types of materials, experimental systems and methods used in many studies. Here, indicate whether each material, system or method listed is relevant to your study. If you are not sure if a list item applies to your research, read the appropriate section before selecting a response.

Materials & experimental systems

n/a	Involved in the study	
[ ]	[x]	Antibodies
[ ]	[x]	Eukaryotic cell lines
[x]	[ ]	Palaeontology
[x]	[ ]	Animals and other organisms
[ ]	[x]	Human research participants
[x]	[ ]	Clinical data

Methods

n/a	Involved in the study	
[x]	[ ]	ChIP-seq
[x]	[ ]	Flow cytometry
[x]	[ ]	MRI-based neuroimaging

Antibodies

Antibodies used

All antibodies used in our studied were listed as following:
NF2, Abcam, ab109244, Rabbit
FGFR2, Abcam, ab109372, Rabbit
JNK, Abcam, ab208035, Rabbit
p-JNK(phospho T183+T183+T221), Abcam, ab124956, Rabbit
p38, Abcam, ab170099, Rabbit
p-p38(phospho T180), Abcam, ab178867, Rabbit
YAP1, Abcam, ab52771, Rabbit
p-YAP1(phospho S127), Abcam, ab76252, Rabbit
p-Rb(phospho S780), Abcam, ab173289, Rabbit

	cyclinD1, Abcam, ab134175, Rabbit<br>Vinculin, Sigma, V9131, Mouse
Validation	All antibodies used in our studied and their validated websites were listed as following:<br>NF2, Rabbit, https://www.abcam.cn/nf2--merlin-antibody-epr25732-ab109244.html#top-550,<br>FGFR2, Rabbit, https://www.abcam.cn/fgfr2-antibody-epr5180-ab109372.html,<br>JNK, Rabbit, https://www.abcam.cn/jnk1jnk2jnk3-antibody-epr18841-95-ab208035.html,<br>p-JNK(phospho T183+T183+T221), Rabbit, https://www.abcam.cn/jnk1--jnk2--jnk3-phospho-t183t183t221-antibody-epr5693-ab124956.html,<br>p38, Rabbit, https://www.abcam.cn/p38-antibody-e229-ab170099.html,<br>p-p38(phospho T180), Rabbit, https://www.abcam.cn/p38-phospho-t180-antibody-epr16587-ab178867.html,<br>YAP1, Rabbit, https://www.abcam.cn/yap1-antibody-ep1674y-ab52771.html,<br>p-YAP1(phospho S127), Rabbit, https://www.abcam.cn/yap1-phospho-s127-antibody-ep1675y-ab76252.html,<br>p-Rb(phospho S780), Rabbit, https://www.abcam.cn/rb-phospho-s780-antibody-epr182n-ab173289.html,<br>cyclinD1, Rabbit, https://www.abcam.cn/cyclin-d1-antibody-epr2241-c-terminal-ab134175-, references.html#top-730,<br>Vinculin, Mouse, https://www.sigmaaldrich.com/catalog/product/sigma/v9131?lang=zh&region=CN.

Eukaryotic cell lines

Policy information about cell lines

Cell line source(s)	The human embryonic kidney HEK293T (293T), MDA-MB-231 and Hs578T cell lines were obtained from the Shanghai Cell Bank Type Culture Collection Committee (CBTCCC, Shanghai, China) in 2014.
Authentication	The identities of the cell lines were confirmed by Shanghai Cell Bank Type Culture Collection Committee (CBTCCC, Shanghai, China) using DNA profiling (short tandem repeat, STR). The cell lines were subjected to routine cell line quality examination (e.g., by morphology and mycoplasma testing) by HD Biosciences every 3 months.
Mycoplasma contamination	No mycoplasma contamination was confirmed.
Commonly misidentified lines<br>(See ICLAC register)	No misidentified cell lines were used.

Human research participants

Policy information about studies involving human research participants

Population characteristics	A total of 1,134 consecutive Chinese patients who were treated at the Department of Breast Surgery at Fudan University Shanghai Cancer Center (FUSCC). Our study included 419 neoadjuvant breast cancer patients, 606 surgical breast cancer patients, and 109 advanced/metastatic breast cancer patients. All patients are female. The average age of all patients in our study is 52.5 (range: 22 - 88).
Recruitment	Patients diagnosed with malignant breast cancer who were willing to participate in the present study were prospectively recruited. Recruiting criteria included: (1) female patients diagnosed with unilateral breast cancer; (2) central pathological examination of tumor specimens performed by the Department of Pathology at FUSCC; and (3) sufficient frozen tissue available for further investigation. Patients with small tumors would fail to get enough tissues to undertake core needle biopsy and target sequencing, and they would be excluded from the study manually which impacted the patient selection and caused higher proportion of patients with higher tumor stages in our study.
Ethics oversight	The study was approved by the FUSCC Ethics Committee, and all the patients provided written informed consent.

Note that full information on the approval of the study protocol must also be provided in the manuscript.
